# Chemovariation and antibacterial activity of extracts and isolated compounds from species of *Ixora* and *Greenea* (Ixoroideae, Rubiaceae)

**DOI:** 10.7717/peerj.6893

**Published:** 2019-05-07

**Authors:** Raveevatoo Buathong, Voradol Chamchumroon, Johann Schinnerl, Markus Bacher, Wichai Santimaleeworagun, Ekaphan Kraichak, Srunya Vajrodaya

**Affiliations:** 1 Department of Botany, Faculty of Science, Kasetsart University, Bangkok, Thailand; 2 The Forest Herbarium, National Parks, Wildlife and Plant Conservation Department, Bangkok, Thailand; 3 Chemodiversity Research Group, Department of Botany and Biodiversity Research, University of Vienna, Vienna, Austria; 4 Institute of Chemistry of Renewable Resources, University of Natural Resources and Life Sciences Vienna (BOKU), Tulln an der Donau, Austria; 5 Department of Pharmacy, Faculty of Pharmacy, Silpakorn University, Nakhon Phathom, Thailand; 6 Novel Antibiotic Compound Project by Pharmaceutical Initiative for Resistant Bacteria and Infectious Diseases Working Group (PIRBIG), Silpakorn University, Nakhon Phathom, Thailand

**Keywords:** Antibacterials, *Ixora*, *Greenea*, Scopoletin, Geniposidic acid

## Abstract

**Background:**

A large number of secondary metabolites can be obtained from plants used for traditional medicine in two related genera (*Ixora* and *Greenea*) in the subfamily Ixoroideae (Rubiaceae), but there are only a few detailed studies on their bioactivities. Therefore, the main goals of this study were to determine the antibacterial activities of lipophilic extracts from plants of some *Ixora* and *Greenea* species native to Thailand, and to isolate some pure compounds from those extracts. Moreover, we compared the occurrence of compounds in different plant parts of samples from different habitats to better understand their variation.

**Methods:**

A total of 56 lipophilic extracts were obtained from the leaves, stem bark, and root bark of eight *Ixora* and two *Greenea* species collected at various locations in Thailand. Isolated compounds were identified using nuclear magnetic resonance. Antimicrobial activities were evaluated against four Gram-positive and nine Gram-negative human pathogenic bacterial strains.

**Results:**

Extracts from *I. javanica, I. nigricans, I. brunonis, and G. montana*, along with isolated scopoletin, exhibited antibacterial activities against Gram-positive methicillin-resistant* Staphylococcus aureus* ATCC 43300, with minimum inhibitory concentration values ranging from 64 to 256 µg/mL. The occurrence of scopoletin, isofraxidin, and geniposidic acid in lipophilic extracts showed some variation among different plant parts and species.

**Conclusions:**

Lipophilic extracts of *Ixora* and *Greenea* species have the potential to be developed as anti-Gram-positive agents, in particular to counter infections of methicillin-resistant *S. aureus* strains. The chemical profiles showed differences between floristic regions but similarity within the same plant parts.

## Introduction

As the resistance of microorganisms to available antibiotics increases, plant sources have become an attractive alternative for new drug discovery (Cowan, 1999). The World Health Organization (World Health Organization (WHO), 2017) has recently published a list of antibiotic-resistant bacteria, of which methicillin-resistant *Staphylococcus aureus* is considered a high-priority pathogen that urgently requires new antibiotic targeting. A number of studies have identified antibacterial activity against *S. aureus* strains in extracts from *Ixora* species (Annapurna et al., 2003; Mani et al., 2014). Furthermore, a large number of species in the genus *Ixora* are used as traditional treatments for many ailments. In folk medicine, species such as *I. brunonis*, *I. cibdela*, *I. javanica*, and *I. nigricans* are used to treat fevers, ophthalmic diseases, ear infections, wounds infections, dysentery, and diarrhea (Table S1). These diseases are mostly caused by bacteria and viruses; therefore, *Ixora* species are potential natural sources for antimicrobial drug development. Moreover, plants in the genus *Greenea*, native to Southeast Asia (Puff, Chayamarit & Chamchumroon, 2005; Tange, 2013), are closely related to *Ixora* according to phylogenetic analysis (Razafimandimbison et al., 2011; Kainulainen, Razafimandimbison & Bremer, 2013). Given this phylogenetic affinity, it is possible that extracts from *Greenea* could have bioactivities similar to those from *Ixora*.

**Table 1:** Distribution of scopoletin (1), isofraxidin (2), and geniposidic acid (3) in leaves, stem bark and root bark extracts from examined *Ixora* and *Greenea* species collected from northeastern and southern Thailand.

Species	Collector No.	Origin	Leaves	Stem bark	Root bark
*I. javanica* (Blume) DC.	RB001	Nakhon Ratchasima	(**1**)	(**1**), (**2**)	NS
	RB002	Nakhon Ratchasima	(**1**)	(**1**), (**2**)	(**1**), (**2**)
	RB021	Chalung, Songkhla	–	(**1**)	NS
	RB023	Phang Nga	NS	(**1**)	(**1**)
	RB025	Thung Tam Sao, Songkhla	(**1**)	(**1**)	NS
	RB028	Thung Tam Sao, Songkhla	(**1**)	(**1**)	NS
	RB041	Trang	(**1**)	(**1**)	(**1**)
	RB042	Trang	(**1**)	(**1**)	(**1**)
	RB024	Phang Nga	(**1**)	(**1**)	NS
*I. cibdela* Craib	RB046	Sakon Nakhon	(**1**), (**3**)	(**1**), (**2**), (**3**)	(**1**), (**2**), (**3**)
	RB047	Sakon Nakhon	NS	NS	(**1**), (**2**), (**3**)
	RB048	Sakon Nakhon	NS	NS	(**1**), (**2**), (**3**)
*I. diversifolia* R. Br. ex Kurz	RB026	Thung Tam Sao, Songkhla	(**1**), (**3**)	(**1**)	NS
	RB027	Thung Tam Sao, Songkhla	(**1**), (**3**)	(**1**), (**3**)	NS
*I. pendula* Jack	RB031	Thung Tam Sao, Songkhla	(**3**)	(**3**)	NS
*I. nigricans* R. Br. ex Wight & Arn	RB020	Chalung, Songkhla	–	–	NS
	RB029	Thung Tam Sao, Songkhla	–	–	NS
	RB030	Satun	–	–	–
	RB004	Chumphon	–	–	NS
*I. grandifolia* Zoll. & Moritzi	RB003	Chumphon	–	–	–
*I. lobbii* Loundon	RB034	Thung Tam Sao, Songkhla	–	NS	NS
*I. brunonis* Wall. ex G. Don.	RB032	Thung Tam Sao, Songkhla	–	(**1**)	(**1**)
*G. montana* Tange	RB011	Chumphon	(**1**), (**2**), (**3**)	(**1**), (**2**), (**3**)	(**1**), (**2**), (**3**)
	RB012	Chumphon	(**1**), (**2**), (**3**)	(**1**), (**2**), (**3**)	(**1**), (**2**), (**3**)
*G. corymbose* Jack (Voigt)	RB022	Chalung, Songkhla	(**1**), (**2**), (**3**)	(**1**), (**2**), (**3**)	NS

**Note:**

NS, not studied; –, none of three compounds detectable; northeastern Thailand: Nakhon Ratchasima, and Sakon Nakhon provinces; southern Thailand: Chumphon, Phang Nga, Songkhla, Trang, and Satun provinces.

In traditional medicine, different plant parts of *Ixora* species are used to treat different symptoms (Table S1). Thus, each plant part might possess different bioactive compounds that allow for different applications. To date, a few phytochemical studies of *Ixora* species have reported the presence of alkaloids, anthraquinones, phenolics, peptides, terpenoids, sterols, and iridoid glycosides (Chen, Zhang & Chen, 2016; Usha, Reginald & Immanuel, 2016). However, while there are several reports on the biological activities of extracts from *Ixora* species, only a handful of studies have identified their bioactive compounds. Similarly, only one phytochemical study of *Greenea* has been reported, which identified saponins, triterpenes and steroids in the leaves of *G*. *corymbosa* (Rahmani et al., 1985). Isolation and identification of bioactive compounds is needed for further determination of the therapeutic potential of these medicinal plants. We hypothesized that extracts and compounds from unstudied *Ixora* and *Greenea* species may have antibacterial effects, and that different plant parts and the localities from which the plants are obtained may affect the occurrence of these compounds.

The main goals of this study were to determine the antibacterial activities of lipophilic extracts from selected *Ixora* and *Greenea* species against selected human pathogenic bacteria strains, and to isolate and identify potentially active compounds in these species. A comparative phytochemical study was also conducted on 56 lipophilic extracts from the leaves, stem bark, and root bark of eight *Ixora* and two *Greenea* species collected from various locations in Thailand, using thin layer chromatography (TLC), high performance liquid chromatography (HPLC), and, in some cases, by isolation. Our study is the first to report the composition and antibacterial activities of extracts from *G. montana*.

## Materials and Methods

### Plant materials

For comparative phytochemical study, 25 specimens from eight species of *Ixora* and two species of *Greenea* were collected from northeastern and southern Thailand and identified by S. Vajrodaya and V. Chamchumroon (Table 1). Voucher specimens were deposited in the herbarium at the Department of Botany, Faculty of Science, Kasetsart University, Bangkok, Thailand. We collected leaves, stem bark, and root bark for each plant. With some species, we could only obtain certain parts from a few specimens due to the plants’ scattered distributions across the landscape. The genus *Greenea* was only represented by two species (*G. corymbosa* and *G. montana*) because the other species reported from Thailand are only available as herbarium specimens or are limited to areas not accessible for collecting (Tange, 2013).

### Extraction

Air-dried parts (0.6–267 g leaf, stem bark, or root bark) of *Ixora* and *Greenea* species were chopped into small pieces, powdered separately using a blender, and then macerated with methanol (20–400 mL) for a week in the dark at room temperature. The methanolic extracts were filtered and concentrated using a rotary evaporator at 37 °C to obtain the semi-solid crude extract, which was subsequently partitioned into hydrophilic and lipophilic extracts using distilled water and chloroform, respectively. The lipophilic and hydrophilic crude extracts were then evaporated to dryness for further experiments. For phytochemical profiling, 10 mg of each extract was prepared in methanol (HPLC grade) and filtered through a 0.45 µm nylon filter prior to analysis by HPLC.

### Identification of chemical profiles

High performance liquid chromatography analysis of lipophilic extracts (10 mg) was performed on an Agilent 1100 series with a UV photodiode array detector at wavelengths of 230, 254, and 280 nm. A reverse phase ChromSepher 5, C18 column (250 × 4.6 mm) was used for analytical separation. The eluent consisted of (A) an aqueous buffer containing 0.015M ortho-phosphoric acid at pH 3 and 0.0015M tetrabutylammonium hydroxide and (B) methanol. The mobile phase started at 60% B for 16 min, then increased to 90–100% B within 6 min, with 100% B continuing for 6 min at a flow rate of 1.0 mLmin^−1^. HPLC analysis of the hydrophilic extract (10 mg) started with the mobile phase at 10% B, then increased to 70% B within 15 min, next to 80% B within 5 min, and finally increased to 100% B within 2 min and continued for 6 min at a flow rate of 0.5 mLmin^−1^. The injection volume was 20 µL for all analyses.

Thin layer chromatography was used to analyze lipophilic extracts on pre-coated silica gel 60 *F*_254_ plates (Merck, Kenilworth, NJ, USA) using a solvent mixture consisting of hexane and ethyl acetate (1:1, v/v). Hydrophilic extracts were analyzed on TLC plates using a solvent mixture of ethyl acetate/methanol/water (6:4:0.4, v/v/v). Compound detection was performed either under UV irradiation or derivatization with anisaldehyde reagent.

### Isolation of compounds

Three compounds were isolated successfully from the following species:

*Ixora javanica* (RB041): The lipophilic extract (130 mg) of stem bark (dry weight 12 g) was separated using column chromatography with a silica gel 60 column (0.04–0.063 mm, Merck, Kenilworth, NJ, USA) with 30% ethyl acetate in hexane. For each fraction, 20 mL was collected. Fractions with blue fluorescence under UV light at 365 nm were combined to obtain compound **1** (10 mg).

*Greenea montana* (RB011): The lipophilic extract (570 mg) of stem bark (dry weight 48 g) was fractionated using a silica gel 60 column (0.2–0.5 mm, Merck, Kenilworth, NJ, USA) with 30% ethyl acetate in hexane. For each fraction, 20 mL was collected. Fractions with slightly dark blue fluorescence under UV light at 365 nm were combined to obtain a mixture of compounds **1** and **2** (2.8 mg).

*Ixora cibdela* (RB046-048): The hydrophilic extract (one g) of root bark (dry weight 26.89 g) was fractionated using Sephadex LH-20 with MeOH as eluent to get ten fractions of 20 mL each (labelled S06). Then, fraction S06-F1 (538 mg) was separated on a silica gel 60 column (0.04–0.063 mm, Merck, Kenilworth, NJ, USA) with increasing polarity of chloroform/methanol mixtures: methanol (at 2:1 v/v) to obtain seven fractions of 30 mL each (labelled SG04). From these, fraction SG04-F1 (12 mg) was subjected to preparative TLC using solvent mixtures of ethyl acetate/methanol/water (7:2:1) to get compound **3** (three mg).

The isolated compounds were identified using nuclear magnetic resonance (NMR) and then used as reference compounds for comparative analyses of different plant parts of *Ixora* and *Greenea* species from different habitats based on retention time and the UV spectra of HPLC profiles.

### Structure elucidation

The structures of the isolated compounds were deduced from 1D- (^1^H, ^13^C) and 2D- (COSY, HSQC, HMBC) NMR spectroscopic data. All NMR spectra were recorded on a Bruker Avance II 400 (resonance frequencies 400.13 MHz for ^1^H and 100.63 MHz for ^13^C) equipped with a five mm observation broadband probe head (BBFO) with *z*-gradients at room temperature and using a standard Bruker pulse program. The samples were dissolved in 0.6 ml of CDCl_3_ or MeOD (each 99.8% D, Euriso-top, Saint-Aubin Cedex, France). Chemical shifts in ppm were determined with reference to residual solvent signals (CDCl_3_: 7.26 ppm for ^1^H, 77.0 ppm for ^13^C, MeO-d4: 3.31 ppm for ^1^H, and 49.0 ppm for ^13^C). The chemical shifts of ^1^H and ^13^C NMR of scopoletin (**1**), isofraxidin (**2**), and geniposidic acid (**3**) are given in the Supplemental File.

### Antibacterial activity

The bacterial strains used in this experiment were methicillin-resistant *S. aureus* (MRSA) ATCC 43300, *S. aureus* ATCC 25923, vancomycin-resistant *Enterococcus faecium* UCLA192, *E. faecalis* ATCC 29212, *Escherichia coli* ATCC 25922, *Pseudomonas aeruginosa* DMST 37166, *P. aeruginosa* ATCC 27853, carbapenem-resistant (KPC-producing) *Klebsiella pneumoniae* ATCC-BAA 1705, *K. pneumoniae* ATCC-BAA 1706, extended-spectrum beta-lactamase (SHV-18 producing) *K. pneumoniae* ATCC 700603, *Acinetobacter baumannii* ATCC 19606, *Stenotrophomonas maltophila* DMST 19079, and *Salmonella choleraesuis* ATCC 10708. These strains were obtained from the Department of Medical Science, Ministry of Public Health, Bangkok, Thailand.

Antibacterial activities were only tested for lipophilic extracts from *I. javanica, I. brunonis, I. nigricans*, and *G. montana*, because these species had complete extract sets including all three different parts of each plant. In addition, isofraxidin could not be isolated as a single pure compound (see isolation of compounds section), and therefore only scopoletin and geniposidic acid were tested.

Disk diffusion assays were used for antibacterial testing of lipophilic extracts, scopoletin, and geniposidic acid. Bacterial strains were inoculated into Muller–Hinton broth (MHB-Difco, Sparks, MD, USA). Bacterial suspensions equivalent in density to 0.5 McFarland (comparable to 1.5 × 10^8^ CFU/mL) were then inoculated on Muller–Hinton agar (MHA-Difco, USA). Plant extracts (400 µg) were dissolved in 100 µL dimethyl sulfoxide (DMSO; Sigma-Aldrich, St Louis, MO, USA), loaded into six mm diameter cylinder cups, and placed on media containing the test organism. Plates were subsequently incubated for 18–20 h at the optimal temperature for the bacterial strain. The resulting inhibition zones (diameter of clear zone; mm) were compared to those of standard antibiotics piperacillin/tazobactam (100/10 µg), amikacin (30 µg), ceftriaxone (30 μg), cefotaxime (30 µg), ceftazidime (30 μg), ciprofloxacin (five µg), nalidixic (30 μg), imipenem (10 μg), trimethoprim/sulfamethoxazole (1.25/23.75 µg), and vancomycin (30 µg) with control species *K. pneumoniae* ATCC 700603 and *P. aeruginosa* strain ATCC 27853, following the recommendation of the Clinical and Laboratory Standards Institute (CLSI, 2017) for quality control of antimicrobial testing (Table S2).

A broth microdilution test was also used to determine the minimum inhibitory concentrations (MICs) of various lipophilic extracts, scopoletin, and geniposidic acid. Serial dilutions of extracts in DMSO, ranging from 1,024 to 0.5 µg/mL, were prepared and placed into 96-well microplates. To these were added a standard inoculum of the tested bacteria in MHB, which was then incubated at 37 °C for 18 h. The MIC was determined from the lowest concentration of extract that showed inhibition of bacterial growth.

## Results

### Occurrence pattern of coumarins and iridoid in the studied *Ixora* and *Greenea* species

Phytochemical examination and isolation of extracts from *Ixora* and *Greenea* species revealed three known compounds: two coumarins, scopoletin (**1**) and isofraxidin (**2**), and one iridoid glycoside, geniposidic acid (**3**). These compounds were identified using NMR spectroscopy. The occurrences of these compounds varied among extracts from different plant parts (leaves, stem bark, and root bark) in species of both genera collected from different provinces (Table 1). All three were detected in all parts of the studied *Greenea* samples. In contrast, these compounds occurred sporadically in different parts of *Ixora* plants. Among *Ixora* species, isofraxidin was found only in stem and root bark extracts of samples collected from northeastern Thailand. Meanwhile, extracts from *I. pendula* did not contain either scopoletin or isofraxidin, making it completely unlike other *Ixora* species.

### Antibacterial activity

**Figure 1:**
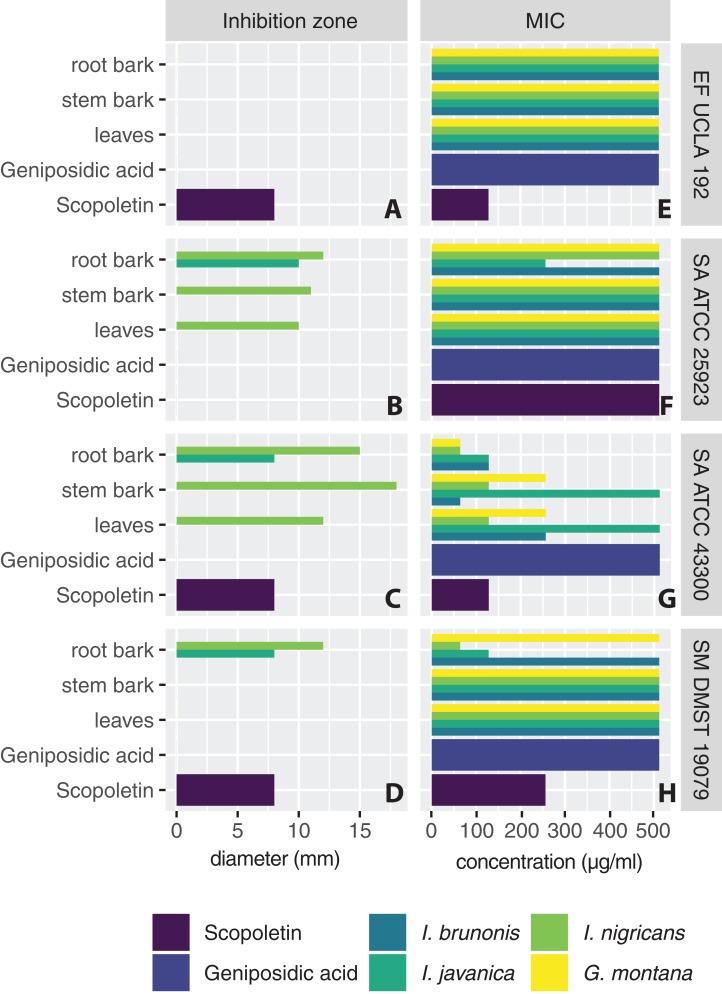
Inhibition zone diameters in mm (A–D) and MIC values in µg/mL (E–H) of studied extracts and isolated compounds. Diameter >6 mm indicates inhibition zone. MIC values at the lowest concentration that inhibited visible growth. (A and E) *Enterococcus faecium* UCLA192 (**EF UCLA192**). (B and F) *Staphylococcus aureus* ATCC 25923 (**SA ATCC 25923**). (C and G) *Staphylococcus aureus* ATCC 43300 (**SA ATCC 43300**). (D and H) *Stenotrophomonas maltophila* DMST 19079 (**SM DMST 19079**).

Lipophilic extracts from *Ixora* and *Greenea* species showed antibacterial activity against three of the tested microorganisms: the Gram-positive bacteria methicillin-resistant *S. aureus* ATCC 43300 and *S. aureus* ATCC 25923 and the Gram-negative bacteria *S. maltophila* DMST 19079. Other microorganisms did not show any susceptibility. The inhibition zone diameters and MIC values of studied extracts against susceptible bacteria strains are presented in Fig. 1. All extracts from *I. nigricans* showed potential antimicrobial activity against two strains of the Gram-positive bacterium *S. aureus*, with zones of inhibition measuring 10–18 mm. For *I. javanica*, only the root bark extract was active, affecting the Gram-positive bacterium *S. aureus* and the Gram-negative bacterium *S. maltophila* DMST 19079; the zones of inhibition measured 8–10 mm. Extracts from *I. brunonis* and *G. montana* showed antibacterial activity only in the microdilution assay.

In microdilution assays, lipophilic extracts from all parts of *I. nigricans*, *I. brunonis*, and *G. montana* and the root bark extract of *I. javanica* showed strong antibacterial activity against the Gram-positive, methicillin-resistant *S. aureus* ATCC 43300, with MIC values varying from 64 to 256 µg/mL. In addition, the lipophilic root bark extract of *I. javanica* also showed antibacterial activities against the Gram-positive *S. aureus* ATCC 25923 (MIC value 256 µg/mL) and against the Gram-negative *S. maltophila* DMST 19079 (MIC value 128 µg/mL).

The inhibition zone diameters and MIC values of isolated scopoletin and geniposidic acid are presented in Fig. 1. Scopoletin showed antibacterial activities against the Gram-positive bacteria *S. aureus* ATCC 43300 and *E. faecium* UCLA192 and the Gram-negative bacterium *S. maltophila* DMST 19079, with similar zones of inhibition at eight mm. In microdilution assays, scopoletin showed antibacterial activities against the Gram-positive bacteria *S. aureus* ATCC 43300 and *E. faecium* (MIC value 128 µg/mL), and also against Gram-negative *S. maltophila* strains (MIC value 256 µg/mL). Geniposidic acid did not show any antibacterial activity.

## Discussion

### Detected compounds

Three known compounds (scopoletin, isofraxidin, and geniposidic acid) were detected and isolated from the extracts of *Ixora* and *Greenea* species. Scopoletin (**1**) is a widely distributed coumarin found in a number of plant families such as Rutaceae and Apiaceae (Jain & Joshi, 2012), Clusiaceae (Marcondes et al., 2015), Convolvulaceae (Sood, Pradhan & Biswasroy, 2014; Khan & Hossain, 2015; Ojha, Mishra & Mishra, 2016), Solanaceae (Valizadeh et al., 2014), Asteraceae (Adams, Efferth & Bauer, 2006), Burseraceae (Mogana, Teng-Jin & Wiart, 2013), Asclepiadaceae (El-Demerdash et al., 2009), and Rubiaceae (Swe, 2008; Mohammed et al., 2013; Chen, Zhang & Chen, 2016). Isofraxidin (**2**) is found in species of Araliaceae (Maruyama et al., 2008; Yamazaki & Tokiwa, 2010; Bai et al., 2011; Lee et al., 2013), Asteraceae (Kwak, Lee & Schmitz, 2001; Gliszczyńska & Brodelius, 2012; Yim et al., 2017), Apiaceae (Yim et al., 2017), Simaroubaceae (Hwang et al., 2012), Rutaceae (Brown et al., 1988), Chloranthaceae (Niu et al., 2012; Li et al., 2016), and Rubiaceae (De Araújo et al., 2012).

Iridoids (especially iridoid glycosides) are reported as one of the most common secondary metabolites in Ixoroideae (Inouye et al., 1988; Martins & Nunez, 2015). In this study, only geniposidic acid (**3**) was isolated from *I. cibdela* and detected in *I. diversifolia*, *I. pendula*, *G. montana*, and *G. corymbosa* collections. Geniposidic acid was reported for the first time in *I. chinensis* in 1975 by Takeda, Nishimura & Inouye, but has not been reported in other species of the genus. Our results reveal that iridoid glycosides also occur in other *Ixora* and *Greenea* species. Similarly, other iridoid compounds may yet be present in our tested species. For example, ixoroside and ixoside were reported in *I. chinensis* (Takeda, Nishimura & Inouye, 1975), asperuloside was reported from *I. casei*, *I. japonica*, and *I. macrothyrsa* (Inouye et al., 1988), and deacetylasperulosidic acid was reported in *I. odorata* (Inouye et al., 1988).

The occurrences of scopoletin, isofraxidin, and geniposidic acid in leaf extracts of the two studied *Greenea* species were entirely different from those in the leaves of the tested *Ixora* species. However, these compounds are not the best candidates for chemotaxonomic markers to distinguish these genera, as they were detected in other parts for specimens of both *Greenea* species collected from the south, as well as in *I. cibdela* collected from the northeast. The presence or absence of secondary metabolites may be caused by a number of factors such as soil, season of collection, habitat, plant organ, plant age, and genetic mutation (Martins & Nunez, 2015). Moreover, the availability of building blocks for the biosynthesis of secondary metabolites and infection by microorganisms also affect metabolite production (Wilart, 2013). Building blocks for secondary metabolites are synthesized from intermediates or end-products of primary metabolic pathways such as photosynthesis, glycolysis, and the Krebs cycle; catalyzing enzymes and cofactors in these pathways can yield various classes of natural products, depending on a sequence of reactions and rearrangement in the biosynthesis pathway (Dewick, 2002; Ribera & Zuñiga, 2012; Gokulan, Khare & Cerniglia, 2014). This suggests that chemical variation among different plant parts and collecting locations may result from different biotic or abiotic factors affecting the accumulation of secondary metabolites.

### Antibacterial activities

The findings of this study were similar to previous work on methanolic leaf and stem extracts of *I. coccinea* that reported broad antibacterial activities, especially against the Gram-positive bacterium *S. aureus* (Annapurna et al., 2003; Mani et al., 2014). However, while extracts from all parts of *I. brunonis* and *G. montana* showed distinct antibacterial activity in MIC assays, no zone of inhibition was observed in disc diffusion assays for these extracts (Fig. 1). This discrepancy may have been caused by the diffusion of extracts into the agar medium of the disc diffusion assay, making their antibacterial activity less potent than when in the microdilution well of the MIC assay (Mani et al., 2014).

Iridoids have been reported to possess a number of biological and pharmacological activities such as neuroprotective, anti-inflammatory, immunomodulator, hepatoprotective, cardioprotective, anticancer, antioxidant, antimicrobic, hypoglycaemia, hypolipidemic, choleretic, antispasmodic, and purgative properties (Tundis et al., 2008). In particular, geniposidic acid has been reported to inhibit the mycelium development of the plant pathogenic fungus *Colletotrichum gloeosporioides* (Luciano, Lima & Silveira, 2010), but the present study did not find evidence of antibacterial activity at concentrations of 0.5–1,024 µg/mL. However, only moderate antibacterial activity has been reported for the related secoiridoid sweroside, with its MIC at the high concentration of 1.0 g/mL (Horn et al., 2001). Therefore, higher concentrations are required for conclusive evaluation of the antibacterial activity of the iridoid glucoside geniposidic acid.

The antibacterial effects of coumarins have been shown to depend on the substitution patterns in their structures. Scopoletin has previously been shown to have antifungal, antibacterial, antioxidant, and anti-inflammatory properties (Gnonlonfin, Sanni & Brimer, 2012; Balouiri, Sadiki & Ibnsouda, 2016). Scopoletin consists of disubstituted simple coumarins (O-CH_3_ at C6 and OH at C7) or hydroxycoumarins, which exhibit greater antibacterial activity against Gram-positive bacteria than Gram-negative bacteria, perhaps due to exclusion by the antibiotic efflux pump mechanism of Gram-negative bacteria (Tegos et al., 2002; Yang et al., 2016). However, other hydroxycoumarins, especially esculetin (OH at C6 and C7) and daphnetin (OH at C7 and C8), have demonstrated enhanced antibacterial activity against Gram-negative bacteria (De Souza, Monache & Smania, 2005).

In our results, scopoletin was not the only active compound identified among the constituents of lipophilic extracts. There could be synergistic effects for compounds in crude extracts, making the combination more effective than any single isolated constituent (Rasoanaivo et al., 2011). Synergism of mixed compounds in lipophilic extracts may be responsible for the antibacterial activity of a crude extract through various mechanisms, such as prevention from degradation by enzymes, effects on cell signaling and transportation, and overcoming multi-drug resistance mechanisms (Gilbert & Alves, 2003). In particular, the *I. javanica* specimen RB041 of showed scopoletin throughout the plant, but its lipophilic extracts from parts other than the root did not exhibit antibacterial activity, while the leaf extract from *I. brunonis* specimen RB032 contained no scopoletin but did exhibit some activity. Therefore, the antibacterial activities of these plants can be ascribed to other groups of compounds such as terpenoids and steroids.

## Conclusions

Our findings show that the lipophilic extracts of *I. javanica*, *I. nigricans*, *I. brunonis*, and *G. montana* from Thailand have potential to be used as antimicrobial agents against Gram-positive methicillin-resistant *S. aureus* strains. Detailed phytochemical investigations found scopoletin, isofraxidin, and geniposidic acid to be prevalent among the studied species. Isolated scopoletin showed anti-bacterial activity, as did several extracts without detected scopoletin, suggesting that scopoletin is not the only active compound in the lipophilic extracts. Further study is required to reveal other possible bioactive ingredients. The occurrence patterns of these compounds among the samples showed some distinction among the genera and species. Further analyses of more specimens from a broader set of species and from different floristic regions are necessary to obtain a more detailed picture of their chemical profiles.

## Supplemental Information

10.7717/peerj.6893/supp-1 Supplemental Information 1 The chemical shifts of ^1^H and ^13^C NMR of isolated compounds. All isolated compounds were elucidated from 1D and 2D Nuclear Magnetic Resonance (NMR) spectroscopy and by comparison with literature data.

10.7717/peerj.6893/supp-2 Supplemental Information 2 Traditional medicinal uses of *Ixora* species. *Ixora* species for antibacterial test selected from their list of traditional uses.

10.7717/peerj.6893/supp-3 Supplemental Information 3 The inhibition zone diameters (mm) of standard antibiotics with control species. NT, not tested; **KP BAA 1705**: *Klebsiella pneumoniae* ATCC–BAA 1705 (KPC-producing; carbapenem resistant strain), **SA ATCC 43300**: *Staphylococcus aureus* ATCC 43300, and **SC ATCC 10708**: *Salmonella choleraesuis* ATCC 10708. KP^*^ **ATCC 700603**: *K. pneumoniae* ATCC 700603 and PA^*^ **ATCC 27853**: *Pseudomonas aeruginosa* strains were used as control species; the clear zone diameter for each control antibiotic was within the quality control ranges set by the CLSI (2017)*. *The quality control ranges set by the CLSI (2017): For *K. pneumoniae* strain ATCC 700603, the clear zone diameter of ceftazidime, cefotaxime, and ceftriaxone is 10–18, 17–25, and 16–24 mm, respectively.

## Additional Information and Declarations

### Data Availability

The following information was supplied regarding data availability: The raw data and code is available in Table 1 and in the Supplemental Files.
