# Supplementary material for: Chemovariation and antibacterial activity of extracts and isolated compounds from species of Ixora and Greenea (Ixoroideae, Rubiaceae)
Source: PeerJ. 2019 May 7;7:e6893. doi: 10.7717/peerj.6893 (PMC6510216; doi:10.7717/peerj.6893)
Supplement: Supplemental Information 1 — All isolated compounds were elucidated from 1D and 2D Nuclear Magnetic Resonance (NMR) spectroscopy and by comparison with literature data. [file peerj-07-6893-s001.docx]

The chemical shifts of ^1^H and ^13^C NMR of isolated compounds.

Scopoletin (**1**): ^1^H NMR (400 MHz, CDCl3): δ 3.96 (s, 3H, OMe), 6.12 (br.s, 1H, OH), 6.27 (d, 1H, *J* = 9.5 Hz, H-3), 6.84 (s, 1H, H-5), 6.92 (s, 1H, H-8), and 7.59 (d, 1H, *J* = 9.5 Hz, H-4); ^13^C NMR (100 MHz, CDCl3): δ 56.4 (OMe), 103.2 (C-8), 107.5 (C-5), 111.5 (C-4a), 113.5 (C-3), 143.2 (C-4), 144.0 (C-6), 149.5 (C-7), 150.3 (C-8a), and 161.3 (C-2) (Khan & Hossian, 2015).

Isofraxidin (**2**): ^1^H NMR (400 MHz. CDCl3): δ 3.94 (s, 3H, OMe-6), 4.09 (s, 3H, OMe-7,8), 6.26 (d, 1H, *J* = 9.6 Hz, H-3), 6.84 (s, 1H) 6.92 (s, 1H), and 7.57 (d, 1H, *J* = 9.6 Hz, H-4). ^13^C NMR (100 MHz, CDCl3): δ 56.43 (OMe), 103.22 (C-8), 107.51 (C-5), 113.48 (C-3), and 143.21 (C-4) (Gao *et al.,* 2013).

Geniposidic acid (**3**): ^1^H NMR (400 MHz. MeOD): δ 5.06 (d, *J* = 7.5 Hz, H-1), 7.21 (d, *J* = 1.2 Hz, H-3), 3.25 (m, H-5), 2.85 (dd, *J* = 16.5, 8.4 Hz, H-6), 2.08 (dd, *J* = 16.5, 7.7 Hz, H-6), 5.77 (br.s, H-7), 2.67 (t, *J* = 7.7 Hz, H-9), 4.30 (br.d, *J* = 14.3 Hz, H-10), 4.18 (br.d, *J* = 14.3 Hz, H-10), 4.71 (d, *J* = 7.9 Hz, Glc-1), 3.22 (dd, *J* = 9.0, 7.9 Hz, Glc-2), 3.39 (m, Glc-3), 3.31 (m, Glc-4), 3.27 (m, Glc-5), 3.84 (dd, *J* = 12.0, 2.1 Hz, Glc-6), and 3.66 (dd, *J* = 12.0, 5.3 Hz, Glc-6). ^13^C NMR (100 MHz, MeOD): δ 97.76 (C-1), 148.37 (C-3), 118.94 (C-4), 37.85 (C-5), 128.50 (C-6), 144.94 (C-7), 47.48 (C-8), 61.67 (C-9), 175.94 (C-10), 100.23 (C-11), 75.00 (Glc-1), 77.85 (Glc-2), 71.57 (Glc-3), 78.27 (Glc-4), 62.64 (Glc-5), and 97.76 (Glc-6) (Tao *et al*., 2007; Yahara *et al*., 1990).

All isolated compounds were elucidated from 1D and 2D Nuclear Magnetic Resonance (NMR) spectroscopy and by comparison with literature data.
