# Supplementary material for: Chemovariation and antibacterial activity of extracts and isolated compounds from species of Ixora and Greenea (Ixoroideae, Rubiaceae)
Source: PeerJ. 2019 May 7;7:e6893. doi: 10.7717/peerj.6893 (PMC6510216; doi:10.7717/peerj.6893)
Supplement: Supplemental Information 2 — Ixora species for antibacterial test selected from their list of traditional uses. [file peerj-07-6893-s002.docx]

**Supplemental Table 1:**

Traditional medicinal uses of *Ixora* species

| **Species** | **Part** | **Condition treated** | **References** |
| --- | --- | --- | --- |
| *I. brunonis* | root | restoration, muscle soreness  fever remedies | *Neamsuvan, Tanthien & Petchboon, 2014*  *Jantarapol, Tanthien & Neamsuvan, 2014* |
| *I. cibdela* | root | phlegm treatment  rheumatoid arthritis | *Tiengburanatam, 1999*  *Chamchumroon, 2004* |
|  | stem | eardrop to kill centipedes | *Tiengburanatam, 1999* |
|  | leaves | anthelmintic treatment | *Tiengburanatam, 1999* |
|  | flower | ophthalmic diseases | *Tiengburanatam, 1999* |
|  | fruit | nasal polyps treatment | *Tiengburanatam, 1999* |
| *I. fusca* | root | increase the amount of  hemoglobin | *Chamchumroon, 2004* |
| *I. grandifolia* | leaves | sprain, eczema, boils, concussions,  wounds, skin ulcer | *Karat et al., 2013* |
| *I. javanica* | root  leaves  flower | restoration  fever remedies  antipyretic  cancer treatment  restoration  cancer treatment | *Neamsuvan, Tanthien & Petchboon, 2014*  *Jantarapol, Tanthien & Neamsuvan, 2014*  *Chamchumroon, 2004*  *Karat et al., 2013*  *Neamsuvan, Tanthien & Petchboon, 2014*  *Karat et al., 2013* |
|  |  |  |  |
|  |  |  |  |
| *I. lobbii* | root | restoration, nosebleed, swelling,  phlegm treatment | *Tiengburanatam, 1999* |
| *I. nigricans* | root | dysentery, diuretics  diarrhea, ear infections, remedy for unconsciousness, vomiting over bleeding | *Chamchumroon, 2004*  *Yusuf, 2009* |
|  | leaves | boils treatment, dysentery, remedy for unconsciousness | *Yusuf, 2009* |

*Ixora* species for antibacterial test selected from their list of traditional uses.
