# Supplementary material for: Chemovariation and antibacterial activity of extracts and isolated compounds from species of Ixora and Greenea (Ixoroideae, Rubiaceae)
Source: PeerJ. 2019 May 7;7:e6893. doi: 10.7717/peerj.6893 (PMC6510216; doi:10.7717/peerj.6893)
Supplement: Supplemental Information 3 — NT, not tested; KP BAA 1705: Klebsiella pneumoniae ATCC–BAA 1705 (KPC-producing; carbapenem resistant strain), SA ATCC 43300: Staphylococcus aureus ATCC 43300, and SC ATCC 10708: Salmonella choleraesuis ATCC 10708. KP* ATCC 700603: K. pneumoniae ATCC 700603 and PA* ATCC 27853: Pseudomonas aeruginosa strains were used as control species; the clear zone diameter for each control antibiotic was within the quality control ranges set by the CLSI (2017)*. *The quality control ranges set by the CLSI (2017): For K. pneumoniae strain ATCC 700603, the clear zone diameter of ceftazidime, cefotaxime, and ceftriaxone is 10–18, 17–25, and 16–24 mm, respectively. [file peerj-07-6893-s003.docx]

**Supplemental Table 2:**

The inhibition zone diameters (mm) of standard antibiotics with control species.

| **Samples** | **KP**  **BAA 1705** | **SA**  **ATCC 43300** | **SC**  **ATCC 10708** | **KP***  **ATCC 700603** | **PA***  **ATCC 27853** |
| --- | --- | --- | --- | --- | --- |
| Piperacillin/tazobactam (100/10 μg) | **28** | **25** | **30** | NT | **28** |
| Amikacin (30 μg) | **23** | **30** | **25** | **20** | **23** |
| Ceftriaxone (30 μg) | NT | NT | **33** | **18** | NT |
| Cefotaxime (30 μg) | NT | **25** | **32** | **17** | NT |
| Ceftazidime (30 μg) | **26** | NT | NT | **13** | **26** |
| Ciprofloxacin (5 μg) | **30** | **30** | **32** | NT | **30** |
| Nalidixic (30 μg) | NT | NT | **27** | NT | NT |
| Imipenem (10 μg) | **22** | NT | **30** | **29** | **22** |
| Trimethoprim/sulfamethoxazole (1.25/23.75μg) | NT | **30** | NT | NT | NT |
| Vancomycin (30 μg) | NT | **18** | NT | NT | NT |

NT: not tested, **KP BAA 1705**: *Klebsiella pneumoniae* ATCC–BAA 1705 (KPC-producing; carbapenem resistant strain), **SA ATCC 43300**: *Staphylococcus aureus* ATCC 43300, and **SC ATCC 10708**: *Salmonella choleraesuis* ATCC 10708. **KP^*^ ATCC 700603**: *K. pneumoniae* ATCC 700603 and **PA^*^ ATCC 27853**: *Pseudomonas aeruginosa* strains were used as control species; the clear zone diameter for each control antibiotic was within the quality control ranges set by the CLSI (2017)*.

*The quality control ranges set by the CLSI (2017): For *K. pneumoniae* strain ATCC 700603, the clear zone diameter of ceftazidime, cefotaxime, and ceftriaxone is 10-18 mm, 17-25 mm, and16-24 mm, respectively.

For *P. aeruginosa* strain ATCC 27853, the clear zone diameter of piperacillin/tazobactam, amikacin, and ciprofloxacin is 25-33 mm, 18-26 mm, and 25-33 mm, respectively.
